# Trajectory-Based Visual Localization in Underwater Surveying Missions

**DOI:** 10.3390/s150101708

**Published:** 2015-01-14

**Authors:** Antoni Burguera, Francisco Bonin-Font, Gabriel Oliver

**Affiliations:** System, Robotics and Vision Group, Department Matemàtiques i Informàtica,Universitat de les Illes Balears, Ctra. Valldemossa, Km. 7.5, 07122 Palma de Mallorca, Spain; E-Mails: francisco.bonin@uib.es (F.B.-F.); goliver@uib.es (G.O.)

**Keywords:** underwater robotics, visual localization, data association, image registration

## Abstract

We present a new vision-based localization system applied to an autonomous underwater vehicle (AUV) with limited sensing and computation capabilities. The traditional EKF-SLAM approaches are usually expensive in terms of execution time; the approach presented in this paper strengthens this method by adopting a trajectory-based schema that reduces the computational requirements. The pose of the vehicle is estimated using an extended Kalman filter (EKF), which predicts the vehicle motion by means of a visual odometer and corrects these predictions using the data associations (loop closures) between the current frame and the previous ones. One of the most important steps in this procedure is the image registration method, as it reinforces the data association and, thus, makes it possible to close loops reliably. Since the use of standard EKFs entail linearization errors that can distort the vehicle pose estimations, the approach has also been tested using an iterated Kalman filter (IEKF). Experiments have been conducted using a real underwater vehicle in controlled scenarios and in shallow sea waters, showing an excellent performance with very small errors, both in the vehicle pose and in the overall trajectory estimates.

## 1. Introduction

### 1.1. Problem Statement

Thanks to recent technological advances, the sub-aquatic world is more accessible for exploration, scientific research and industrial activity. At present, remotely-operated vehicles (ROVs) are commonly used in a variety of applications, such as surveying, scientific sampling, rescue operations or industrial infrastructure inspection and maintenance.

Trying to overcome some of the intrinsic limitations of ROVs, such as their limited operative range or the need for a support vessel, autonomous underwater vehicles (AUVs) are progressively being introduced, especially in highly repetitive, long or hazardous missions. Because they are untethered and self-powered, AUVs offer a significant independence from support ships and weather conditions, thus reducing notably the operational costs and the complexity of human and material resources, compared to operations conducted with ROVs.

Localization, which consists of determining and keeping track of the robot location in the environment, becomes a crucial issue in AUVs. The mission success depends, to a great extent, on the precision of the estimated vehicle pose. Errors in orientation generate important drifts on the computed robot trajectory, thus hindering the accomplishment of the programmed mission.

There are several ways to estimate the robot motion in underwater vehicles, for instance: (1) using inertial sensors, such as gyroscopes and accelerometers; (2) using odometry, computed via cameras or acoustic sensors, such as sonars or Doppler velocity log (DVL); and (3) combining inertial sensors and odometers, fusing all of the sensorial data by means of navigation filters, such as the extended Kalman filter (EKF) or particle filters, to smooth trajectories and errors ([1–3]).

Nevertheless, all of these measurements are, to a greater or lesser extent, prone to drift, it being necessary to adjust periodically the pose of the vehicle to reduce, as far as possible, the accumulated error. To this end, the so-called simultaneous localization and mapping (SLAM) [4] techniques constitute the most common and successful approach to perform precise localization. The principal aim of SLAM is the reduction of errors present in odometry by localizing the robot with respect to landmarks or significant points of the environment. This localization process is reinforced by recognizing regions previously visited by the robot in a process known as loop closing. Landmarks are incorporated into an incremental map, and their location is refined simultaneously with the vehicle pose.

The process of sensing underwater environments becomes particularly complex. When light propagates in water, it interacts with molecules and dissolved particulate matter. As a consequence, the light traveling distance underwater is dramatically reduced when compared to air. Contrarily, sound propagates faster, and it is able to travel larger distances in water than in air. Consequently, acoustic sensors have been traditionally considered the best choice for underwater vehicles [5–8]. However, acoustic sensors have low spatial and temporal resolutions compared to optical sensors. This means that, in general, they capture less details and scan at lower frequencies than modern cameras with high resolutions and fast frame rates. Thus, although the quality of optical images in sub-aquatic environments is strongly limited by the water and by the illumination conditions, optical cameras offer advantages over acoustic sensors in several scenarios [9]. Visual platforms are not really appropriate in the water column, where it can be difficult to see the seabed or other reference points. However, for surveying or intervention applications, where the vehicle has to navigate relatively close to the sea bottom or it has to locate itself near the object to be manipulated, the use of cameras can be a suitable option. This paper is focused, precisely, on this kind of application.

Lately, researchers have been focusing their efforts on the enhancement of visual localization techniques to be applicable in sub-aquatic environments and to be operative online, in missions conducted by real underwater vehicles.

### 1.2. Related Work

Visual localization in natural sub-aquatic scenarios has several difficulties not present in land: the light attenuation, flickering, scattering and the special nature of underwater environments with no man-made structured frameworks are some of the most important. Under these conditions, it is particularly difficulty to define, find and track reliable features or natural landmarks that can be used to match scenes visualized from different viewpoints and time instants.

The key of a successful underwater visual SLAM lies in the data association procedure (also known as image registration) to detect loop closings. This data association has to be robust under different viewpoints and illumination conditions. The image registration is in charge of recognizing scenes visualized by the robot from different viewpoints, in frames that have certain overlapping or even differences in illumination conditions, and to compute the camera relative displacement between both views.

The literature is scarce in efficient visual SLAM solutions especially addressed to underwater and tested in field robotic systems. Most of these solutions particularize the approach commonly known as EKF-SLAM [4], correcting the dead-reckoning data with the results of an image registration process in an EKF context. These systems normally incorporate newly-observed visual landmarks in a state vector that contains also the vehicle pose and velocity. One of the positive issues of this approach is the continuous correction of the vehicle and all of the landmark poses contained in the filter at every iteration, which involves a simultaneous refinement of the vehicle trajectory and of the whole map.

For example, in [10], this approach is used for an AUV to inspect a ship hull. In this study, the 3D landmarks corresponding to points in the hull are computed from stereo images and included in the EKF state vector. Furthermore, [11] uses a similar approach to improve the DVL pose estimates by means of stereo imagery. In this case, images are pre-processed to enhance their contrast and brightness and, thus, facilitate the landmark detection process.

However, the computational complexity of updating the covariance matrix in EKF-based solutions is *O*(*n*^2^), *n* being the number of landmarks in the state vector. Because of that, including all of the observed visual landmarks in the state vector leads to an excessive computational cost quickly and makes the method useless in mid- and large-scale environments.

In addition to the computational cost problem, another concern for researchers has been how to make their approaches robust to linearization errors, which are inherent to EKF-based methods. For example, in [12], a submapping EKF-SLAM is adopted and tested on an AUV with highly convincing results.

Trying to overcome these problems, some authors take advantage of the structure of the SLAM problem and use the extended information filter (EIF) [13], which is said to be the dual of the EKF, alleviating some of the aforementioned problems.

Other authors adopted different approaches, such as delayed state filtering (DSF). For example, [14] predicts the robot poses by means of dead reckoning and subsequently incorporates them into the state vector. The detection of image overlapping provides pose constraints, which are used to correct the robot trajectory stored in the state vector. In this case, since the visual landmarks are not included in the filter state vector, the computational cost is drastically reduced. Nonetheless, the image registration is still costly. Recent studies in real-time visual SLAM, such as [15], address this problem by carefully selecting informative images in terms of the used visual SLAM map and using a bag-of-words measure to help in the keyframe selection.

Other authors have approached the underwater visual SLAM [16] problem from the graph-optimization [17] or bundle-adjustment point of view. Using these methods, the successive odometric poses of the vehicle and, in some cases, the position of landmarks constitute the subsequent nodes of a graph linked by edges, which usually represent the distance from node to node. When a loop is closed, the complete graph is optimized, which means a complete graph adjustment, entailing nodes (their labels) and distances between them. This approach eludes the linearization errors, but graphs grow hugely with the amount of landmarks incorporated into the map, thus increasing the computational resources needed.

This paper describes our proposal for EKF-based visual localization, which is mainly addressed to mid-scale surveying underwater missions in shallow waters or in environments with a limited space for maneuvering (for instance, monitoring nuclear storage ponds [18]), using low-cost mini or micro-AUVs with limited computational resources. The proposed approach integrates the data provided by different sensors in an efficient manner to elude the aforementioned problems and to provide accurate pose estimates in real time.

Overall, the advantages of this system can be summarized as follows:

- A trajectory-based schema [8,19] is adopted in order to abate EKF linearization errors. Relative AUV displacements are stored in the filter state, similarly to on-line mosaicking [20], but contrarily to other DSF approaches that store absolute robot poses. Using a chain of relative motions instead of the absolute poses makes the trajectory-based approach able to explicitly correct the whole portion of the trajectory connecting a pair of registered images instead of only correcting its end-points, as happens with other DSF methods.
- The proposed trajectory-based schema has the additional advantage of leading to smaller linearization errors than other delayed-state approaches. In most delayed-state approaches, each item in the state vector represents an AUV pose with respect to a global reference frame. Contrarily, in the trajectory-based approach, each item in the state vector represents the AUV pose with respect to the previous robot pose. Thus, covariances in trajectory-based approaches are likely to be significantly smaller than those of delayed-state approaches. Therefore, the trajectory-based approach leads to smaller linearization errors, because the EKF linearization error is tightly related to the covariances: the larger the covariances involved, the larger the linearization error. Furthermore, as the trajectory-based approach holds the covariance for each relative motion, computing the covariance from one point to another introduces less linearization error than doing the same process using global poses, because this last situation involves an inversion transformation and, again, larger covariances. Computing such a covariance is mandatory every time a loop is closed, and thus, loop closings benefit from our proposal.
- Assuming that the distance from the vehicle to the sea bottom is known by other means, the visual data association process is reduced to a 2.5D problem. The computational cost and complexity is significantly reduced with respect a pure 3D approach, since the dimensionality of the problem is smaller and no stereo matching is required. Although the time reduction may not be important on a desktop computer, it is crucial when dealing with AUVs with limited computing resources and shared with other processes that have real-time constraints.
- The process of image registration is, on the one hand, reinforced by using a RANSAC-based algorithm and, on the other, accelerated by applying it only between images that satisfy a certain geometric criteria.

The whole proposal has been tested with AUVs in real underwater environments and also under simulation.

The paper is structured as follows: Section 2 explains the data association and image registration procedure used to detect loop closings. Section 3 details the design and the structure of the EKF used to perform the visual SLAM. Section 4 shows extensive experimentation that validates our approach, and finally, Section 5 concludes the paper and outlines some forthcoming work.

## 2. Image Registration

In applications, such as SLAM, or in topological navigation, data association refers to the registration of current sensory input to previously-gathered data. This process permits identifying parts of the environment already visited by the robot. Registering successfully such pieces of information is essential to perform loop closures, which impose strong pose constraints that increase accuracy in the incremental localization process.

When using visual sensors, data association is tightly related to image registration, which consists of comparing images taken at different instants from different viewpoints and determining if they overlap. If so, the image registration procedure estimates the motion of the camera between the points at which the images where taken, so that they can be represented with respect to a common coordinate frame. In the context of visual localization, these motion estimates provide pose constraints that can be used to improve the AUV pose estimates.

In the context of this study, image registration is always performed between two images. One of them is the most recently gathered, whilst the other is one that was previously gathered and stored.

Common approaches to image registration are based on feature extraction and matching. Generally speaking, if two images overlap, the features in the overlapping region should have similar descriptors, and thus, the feature correspondences could be used to estimate the relative motion between the images. However, when dealing with underwater imagery, some problems arise that are not present in its terrestrial counterparts.

**Figure 1.**
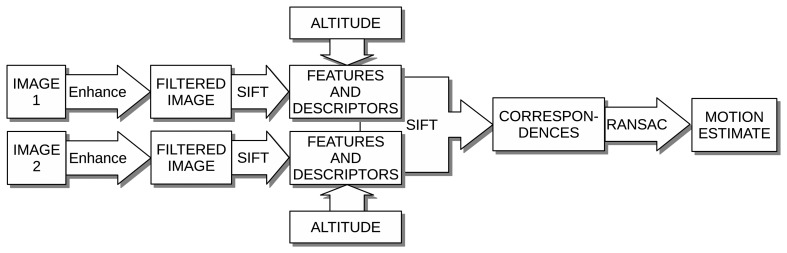
Summary of the proposed image registration process.

For example, due to the light absorption, the underwater image contrast is significantly reduced, leading to poorly descriptive features. In order to achieve reliable and robust feature-based image registration, additional processes are necessary. Our proposal to deal with the aforementioned problems is summarized in Figure 1 and outlined next.

Firstly, the two images under comparison are enhanced with a Butterworth high pass filter. Image regions with low intensity variations, which are mainly due to uneven illumination, vanish, whilst regions with high intensity variations, which are likely to correspond to actual objects, remain.

**Figure 2.**
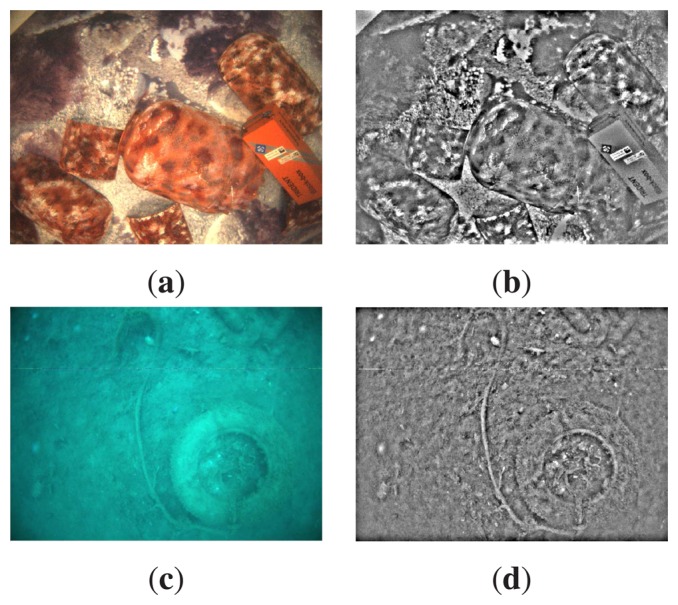
Image processing prior to the image registration. (**a**,**c**) Original images; (**b**,**d**) filtered images.

As an example, Figure 2a,c shows the underwater images gathered by our robot, whilst Figure 2b,d shows the same images after applying the aforementioned filter. As can be observed, image regions with high intensity changes are enhanced and mainly correspond to actual objects in the scene.

Secondly, visual features are searched in the enhanced images. Since changes in orientation, scale and illumination between the two images are expected, scale-invariant feature transform (SIFT) [21] features were used due to their robustness in front of these changes.

At this point, the coordinates of the image features are expressed in pixels. In order to express them in meters, so that they can be properly fused with other sensors' data, the vehicle altitude has to be taken into account. By means of the vehicle altitude and the camera intrinsic parameters and assuming the camera points nadir to a fairly flat sea bottom area, this conversion is straightforward. The result of this conversion is that the 2D features found in the images are projected as 2D points to the locally-flat sea floor. In general underwater surveying missions, the camera orientation assumption is easily affordable, as the vehicle tends to be stabilized in roll and pitch. As for the flat floor assumption, some experiments involving both flat and non-flat terrains will be conducted to see how these influence the results.

**Figure 3.**
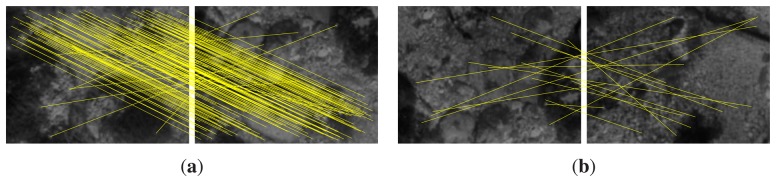
Feature matching using underwater images. Yellow lines represent correspondences between features. (**a**) Overlapping images; (**b**) non-overlapping images.

Finally, features of both images under comparison are matched using the SIFT matcher. At this point, the obtained correspondences could be used to compute the 2D motion between the two feature sets. However, the problems due to directly using feature matching are magnified in these kind of scenarios with which we deal. As an example, Figure 3a shows two images of the same environment where the second one is shifted vertically with respect to the first one. It can be observed that, although most of the SIFT feature matchings, shown as yellow lines, seem to properly capture the motion, some of them do not. If the relative motion between the images is computed using this information, the wrong correspondences would introduce some error in the estimate. Figure 3b shows another example in which the two images correspond to completely different areas that do not overlap. However, SIFT wrongly finds some correspondences. In both cases, the wrong correspondences are said to be outliers, whilst the correct ones constitute the inliers.

In order to find the relative motion between the images that takes into account the inliers and discards the outliers, our proposal is to use random sample consensus (RANSAC) [22]. Thanks to this RANSAC-based approach, our method is able to determine if two images actually overlap and, only in case they do, to provide a 2D motion estimate between them.

Although the RANSAC-based algorithm here proposed is mostly coincident with the general RANSAC approach, it is shown in Algorithm 1 for the sake of completeness. The symbol ⨁ denotes the compounding operator, as described in [23].

The algorithm relies on the so-called *find*_*motion* function, which takes a set of feature matchings *C* and their coordinates in the first (*F_ref_*) and in the second image (*F_cur_*) as inputs. This function provides the 2D roto-translation *X* that better explains the overlap between the images by searching the values of *x*, *y* and *θ* that minimize the sum of squared distances between the matchings in *C*. More specifically, the roto-translation *X* and the associated error *ε* are computed as follows:

(1) $$X=\underset{rt}{\arg\min}f(rt)$$

(2) ε=f(X)

being:

(3) $$f(rt)=\sum_{\forall \left(i,j\right)\in C}{\left\|p_{i}-rt⊕q_{j}\right\|}^{2}$$

where *p_i_* and *q_j_* are feature coordinates in *F_ref_* and *F_cur_*, respectively, and *rt* is the roto-translation that transforms *q_j_* into *p_i_*.



**Figure 4.**
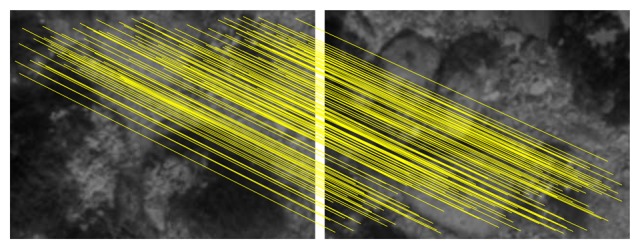
RANSAC underwater image registration using 2D features.

**Algorithm 1:** RANSAC image registration. If the images can be registered, the output *X_best_* = (*x*, *y*, *θ*) is the 2D motion between the two registered feature sets previously projected to the sea floor.
**Input:***F_ref_*: Features {*p*_1_,*p*_2_, ⋯,*p_m_*} in the first image projected to the sea floor*F_cur_*: Features {*q*_1_,*q*_2_, ⋯,*q_n_*} in the second image projected to the sea floor*M*: Matchings *M* = {(*i*,*j*)|*visual*_*matching*(*p_i_*,*q_j_*)}*nIter*: Number of iterations to perform*N*: Number of matchings to be randomly selected*α*: Maximum allowable error per matching*β*: Min. number of selected matches to consider a model**Output:***X_best_*: The estimated 2D roto-translation*ε_best_*: The error of the estimated roto-translation*found*: Boolean stating if reliable matching found**Algorithm:****begin** *k* ←0 ; *ε_best_* ← ∞; *found* ← *false*; **while**
*k* < *nIter*
**do**  *C* ← random selection of *N* items from *M*;  (*X*, *ε*) ← find_motion(*F_ref_*, *F_cur_*, *C*);  **foreach** (*i*,*j*) ∈ (*M* − *C*) **do**   **if** ∥*pi* –*X* ⨁ *q_j_*∥ < *α*
**then**    *C* ← *C* ∪ {(*i*,*j*)};  **if** |*C*| > *β*
**then**   (*X*, *ε*) ← find_motion(*F_ref_*, *F_cur_*, *C*);   **if**
*ε* < *ε_best_*
**then**    *ε_best_* ← *ε* ; *X_best_* ← *X* ; *found* ← *true*;  *k* ← *k* + 1;


As an example, Figure 4 shows the feature correspondences after applying our proposal to the images previously shown in Figure 3a. It can be seen how the wrong correspondences have been rejected and only those explaining the true motion remain. Our proposal has also been applied to the images in Figure 3b, determining correctly that they do no overlap.

The most time-consuming parts of this process are RANSAC and the feature detection. Further sections will discuss how to reduce the number of RANSAC executions. As for the feature detection, it is important to emphasize that they have to be computed only once per image. That is, although the image registration is performed between two images, only the features corresponding to the most recently gathered images have to be computed, as the features corresponding to previously gathered ones have already been extracted in previous time steps.

## 3. Visual Localization

Our proposal to estimate the AUV pose is to combine the dead reckoning estimates with the pose constraints provided by the image registration. Registering each of the gathered images with the current one is an extremely time-consuming task, as cameras usually provide tenths of frames per second. To avoid this problem, in this paper, only one every N frames is registered. Henceforth, N will be referred to as keyframe separation, and each of the registered images will be referred to as keyframes.

A trajectory-based EKF-SLAM schema is used to fuse the above-mentioned sources of information. Being based on EKF, the localization process is performed in three steps: prediction, state augmentation and update. The dead reckoning information and the relevant information of each keyframe are used in the prediction and state augmentation steps. Finally, the pose constraints due to image registration are used in the update step.

The key aspects of the trajectory-based schema, particularized in an EKF implementation, can be summarized as:

- The state vector consists entirely of delayed vehicle states corresponding to each keyframe, contrary to classical approaches, where landmarks are stored in the state vector. This variant offers an important reduction in computational complexity, similar to the DSF approach.
- Instead of storing the absolute robot poses in the state vector, as is commonly done in DFS, the trajectory-based approach stores relative motions between consecutively gathered keyframes.
- Pose samples are refined by incorporating the constraints imposed by the image registration.

The state vector *X_k_* is defined as follows:

(4) $$X_{k}={\left[{\left(x_{1}^{0}\right)}^{T}{\left(x_{2}^{1}\right)}^{T}\ldots {\left(x_{k}^{k-1}\right)}^{T}\right]}^{T}$$

where each $x_{i}^{i-1}\left(2\leq i\leq k\right)$ denotes a roto-translation from keyframe *F_i_*_−1_ to keyframe *F_i_* and $x_{1}^{0}$ represents the initial robot pose relative to a world fixed coordinate frame. It can be assumed, without loss of generality, that $x_{1}^{0}={\left[0,0,0\right]}^{T}$.

The relative pose $x_{j}^{i}$ between two arbitrary keyframes can be computed from the state vector as follows:

(5) $$x_{j}^{i}=\{\begin{array}{ll} \overset{j-1}{\underset{k=i}{⊕}}x_{k+1}^{k} & j>i\\ \overset{i-j}{\underset{k=1}{⊕}}\left(⊖x_{i-k+1}^{i-k}\right) & j<i\\ {\left[0,0,0\right]}^{T} & j=i \end{array}$$

where ⊖ denotes the standard inversion operator [23]. In particular, the pose of a keyframe *j* with respect to the world fixed coordinate frame can be computed as $x_{j}^{0}$. Furthermore, the current robot pose can be computed by composing the last keyframe pose estimate and the dead reckoning information.

### 3.1. Prediction and State Augmentation

Every time a new node *k* is available, both prediction and state augmentation are executed. Assuming a static environment, the state vector does not change during the prediction, and thus, nothing has to be done. As for the state augmentation, the relative motion between keyframe *k* − 1 and keyframe *k* is included in the state vector as follows:

(6) $$X_{k}^{-}={\left[{\left(X_{k-1}^{+}\right)}^{T}{\left(X_{k}^{k-1}\right)}^{T}\right]}^{T}$$

where $x_{k}^{k-1}$ comes from dead reckoning. The term $X_{k-1}^{+}$ denotes the updated state vector corresponding to the previous keyframe. As has been said, the state vector does not change during the prediction step.

Relevant keyframe information, other than the associated relative motion, is stored externally to the state vector. In particular, as suggested in Section 2, the keyframe SIFT features and descriptors are stored externally to the state vector.

### 3.2. The Update Step

During the update step, the pose constraints, given by the overlaps detected after registering candidate images with each current frame, are used to correct the predicted state vector.

#### 3.2.1. Image Overlapping

In order to detect loop closings, every time a new keyframe is gathered, it could be compared with all of the previous ones using the image registration algorithm proposed in Section 2. However, performing such an exhaustive test at every filter iteration can be extremely time consuming.

Our goal is to execute the proposed RANSAC-based image registration only with images that are likely to overlap. In this way, the execution time will be significantly reduced, and in addition, the overall localization accuracy will be improved. Different approaches can be found in the literature concerning this issue [24]. For example, [25] takes into account only highly informative loop closures and non-redundant poses.

Given that one of the goals of this study is to provide SLAM capabilities to small AUVs with reduced computational resources, our priority at this point is the execution speed. That is, the process to decide whether two images overlap sufficiently to execute the time consuming image registration has to be fast. The proposal in this paper is to adopt a purely geometric approach.

As using geometry to detect loop closures assumes good pose estimates, the geometric criteria may lead to wrong guesses about which images register. This means that some images that do not overlap are selected as candidates (false positives) and that images that do overlap are not selected as candidates (false negatives).

False positives can only be responsible for an increase in the computation time, as the RANSAC rejects them during the registration. However, false negatives have the effect of losing the opportunity to improve the pose estimate, thus leading to over-confident estimates [25].

Nevertheless, taking into account the kind of scenarios to which our approach is addressed, neither false positives nor false negatives actually jeopardize the localization process; on the one hand, because their effects will only be appreciable when closing loops after accumulating significant pose error. This is not likely to happen, as small loop closures involving some consecutively gathered images happen very frequently, strongly reducing the odometric error. On the other hand, it has to be taken into account that our proposal targets low-cost AUVs with reduced computational capabilities moving in small-to-mid-scale scenarios. Taking into account both considerations (frequent small loop closures and small-to-mid-scale scenarios), odometric error is not likely to grow enough to produce significant amounts of false positives or negatives. Moreover, even in these cases, the pose estimate is not going to be compromised by false positives due to the robust RANSAC rejection criteria. Of course, if our approach is to be used in large-scale environments, other candidate selection criteria should be used.

**Figure 5.**
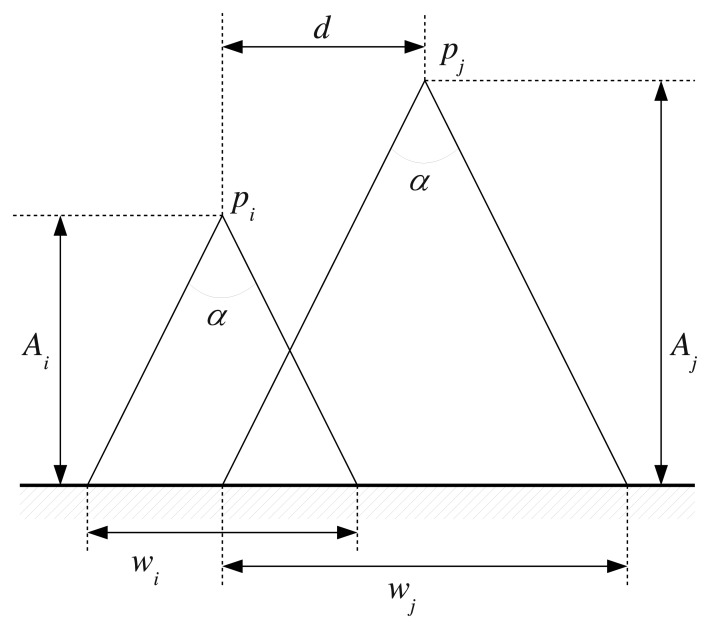
Simple camera model to determine whether two images overlap or not. Given two images gathered at times *t_i_* and *t_j_* and heights *A_i_* and *A_j_* using a camera with an angle of vision of *α* degrees, the observed regions have a diameter of *w_i_* and *w_j_*, respectively. The term *d* denotes the distance between the image acquisition points.

The used geometric criteria are described next. The camera field of view can be modeled as a cone. Under this assumption, the region of the sea bottom observed by the camera is a circle, whose radius depends on the lens field of view and the height at which the image is taken. The field of view being constant, the observed region basically depends on the camera's height when the image is obtained. Accordingly, it can be decided whether two images overlap or not using the height information and the position at which they were gathered. This idea is illustrated in Figure 5.

It is immediate that the diameter of the observed region is as follows:

(7) $$\begin{array}{cc} w_{k}=2\cdot A_{k}\cdot \tan\left(\frac{\alpha }{2}\right) & (k=i,j) \end{array}$$

Accordingly, two images gathered at times *t_i_* and *t_j_* may overlap if the following condition is satisfied:

(8) $$\left\|p_{i}-p_{j}\right\|\leq \frac{w_{i}}{2}+\frac{w_{j}}{2}=\left(A_{i}+A_{j}\right)\cdot \tan\left(\frac{\alpha }{2}\right)$$

where *p_i_* and *p_j_* denote the camera position at times *t_i_* and *t_j_*, respectively, and can be obtained from the state vector.

However, these criteria tell us that two images may overlap if their corresponding vision cones overlap. In real applications, it is desirable to require a larger intersection region. Otherwise, some of the image pairs selected by these criteria would be rejected later by RANSAC, unnecessarily spending computational resources. Our proposal is to include a scaling factor to Equation (8), so that two images may overlap if the following condition is met:

(9) $$\left\|p_{i}-p_{j}\right\|\leq d_{\max}\left(i,j\right)=R\cdot \left(A_{i}+A_{j}\right)\cdot \tan\left(\frac{\alpha }{2}\right)$$

where *R*, which is the scaling factor, should have values between zero and one to increase the required intersection area. Using *R* = 1 means that all possible candidates are considered from the geometrical point of view, but also that false positives are possible. False positives will not affect the quality of the pose estimates, but only increase the computation time, as they will be rejected by RANSAC. Furthermore, even using *R* = 1, some overlapping images may not be considered as candidates. That is, some false negatives may appear, because the geometric criteria are affected by the errors in the pose estimates. Nonetheless, as stated previously, the expected number of false negatives is small, and so, their effect is almost neglectable, as evidenced by the experimental results. The value of *R* can be tuned experimentally, as will be shown in Section 4.

Overall, when a new keyframe *k* is gathered, it is compared with any preceding one if satisfying Equation (9). Evaluating this condition is extremely fast, thus leading to a huge reduction in computation time thanks to the proper choice of image couples to which the RANSAC-based motion estimation is applied.

Moreover, if the robot is moving at a constant height *A_n_*, then *d_max_* is constant, and Equation (9) can be reformulated as:

(10) $$\left\|p_{i}-p_{j}\right\|\leq d_{f}=R\cdot d_{\max}\left(i,j\right)=R\cdot 2\cdot A_{n}\cdot \tan\left(\frac{\alpha }{2}\right)$$

Common AUV applications, where robots have to survey an area for mapping, object detection or intervention, are usually performed at a constant height. Moreover, if these missions are performed in calm waters, AUV controllers are able to maintain a constant height with low error. In these cases, this approach, only involving a single threshold *d_f_*, can be used.

To conclude, if altitude information is available, the function *d_max_*(*i*,*j*) in Equation (9) can be used to determine a possible overlap between the two images gathered at poses *i* and *j*. If no altitude information is available, but the vehicle navigates mostly at a constant height, then the constant threshold *d_f_* defined in Equation (10) is the best choice. In both cases, the pairs of images selected using this method are those that are registered by means of the aforementioned, RANSAC-based approach.

### 3.2.2. Data Associations as a Measurement Vector

Data association is in charge of comparing images, determining if they overlap and, if they do, computing the roto-translation that better explains the overlap. In the context of this paper, this information is used to build the measurement vector *Z_k_*:

(11) $$Z_{k}={\left[{\left(z_{k}^{C1}\right)}^{T},{\left(z_{k}^{C2}\right)}^{T},\ldots ,{\left(z_{k}^{Cn}\right)}^{T}\right]}^{T}$$

where *C*1, *C*2, ⋯,*Cn* denote the keyframes that match the most recent one. The term $z_{k}^{C_{i}}$ represents the motion from keyframe *C_i_* to that most recently gathered according to the image registration described in Section 2.

The observation function *h_i_* outputs an estimation of $z_{k}^{C_{i}}$ according to the state vector $X_{k}^{-}$. As the state vector stores relative motions between keyframes, this can be computed as follows:

(12) $$h_{i}\left(X_{k}^{-}\right)=x_{C_{i+1}}^{C_{i}}⊕x_{C_{i+2}}^{C_{i+1}}⊕\ldots ⊕x_{k}^{k-1}$$

**Figure 6.**
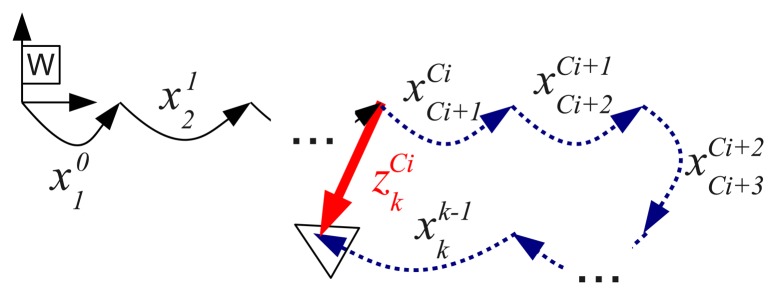
Illustration of a measurement (thick red arrow) and the corresponding observation function (dashed blue arrows)

Figure 6 illustrates the idea of a measurement $z_{k}^{C_{i}}$ and the associated observation function *h_i_*. The observation matrix *H_i_* is as follows:

(13) $$H_{i}={\frac{\partial h_{i}}{\partial X_{k}}|}_{X_{k}^{-}}=\left[{\frac{\partial h_{i}}{\partial x_{1}^{0}}|}_{X_{k}^{-}}{\frac{\partial h_{i}}{\partial x_{2}^{1}}|}_{X_{k}^{-}}\ldots {\frac{\partial h_{i}}{\partial x_{k}^{k-1}}|}_{X_{k}^{-}}\right]$$

It is straightforward to see that:

(14) $$H_{i}=\left[\begin{array}{llll} \underset{\times C_{i}}{\underset{︸}{\begin{array}{c} 000\\ 000\\ 000 \end{array}}} & {\frac{\partial h_{i}}{\partial x_{C_{i+1}}^{C_{i}}}|}_{X_{k}^{-}} & {\frac{\partial h_{i}}{\partial x_{C_{i+2}}^{C_{i+1}}}|}_{X_{k}^{-}}\ldots & {\frac{\partial h_{i}}{\partial x_{k}^{k-1}}|}_{X_{k}^{-}} \end{array}\right]$$

As can be observed, all of the terms in *H_i_* related to the sequence of movements in the measured loop closing will be non-zero. Because of that, contrary to DSF methods [26], the trajectory-based approach leads to non-sparse observation matrices, and thus, it may not scale well for large environments. However, it has to be taken into account that the point at which the environment can be considered large (*i.e.*, when the non-sparsity becomes noticeable) depends on the keyframe separation. Furthermore, the trajectory-based approach reduces the EKF linearization errors, not only with respect to classical EKF methods, but also with respect to traditional DSF-based approaches. The reasons for this reduction have been summarized in Section 1.2. As a matter of fact, the trajectory-based approach surpassed traditional and DSF-based methods in significantly large environments [8] using noisy acoustic sensors. For these reasons, regardless of the non-sparsity of *H_i_*, the trajectory-based approach is one of the best choices for low- and mid-scale surveying missions, like the ones with which this research deals.

Using the analysis from [27], Equation (14) can be computed as follows:

(15) $${\frac{\partial h_{i}}{\partial x_{j}^{j-1}}|}_{X_{k}^{-}}={J_{1⊕}\left\{g_{j},⊖g_{j}⊕h_{i}\right\}|}_{X_{k}^{-}}\cdot {J_{2⊕}\left\{g_{j}⊕\left(⊖x_{j}\right),x_{j}\right\}|}_{X_{k}^{-}}$$

where *J*_1⨁_ and *J*_2⨁_ are the Jacobians of the composition of transformations [23] and:

(16) $$g_{j}=x_{C_{i+1}}^{C_{i}}⊕x_{C_{i+2}}^{C_{i+1}}⊕\ldots ⊕x_{j}^{j-1}$$

At this point, the full observation function *h* and the full observation matrix *H*, considering all of the matched keyframes, are as follows:

(17) $$\begin{array}{cc} h\left(X_{k}^{-}\right)=\left[\begin{array}{c} h_{1}\\ h_{2}\\ \cdots \\ h_{n} \end{array}\right] & H= \end{array}\left[\begin{array}{c} H_{1}\\ H_{2}\\ \cdots \\ H_{n} \end{array}\right]$$

In brief, the observation function estimates the relative position between two overlapping frames composing all of the intermediate displacements stored in the state vector, while the measurement vector stores the relative position between the same overlapped frames directly obtained from the image registration algorithm. The difference between both values, which is the so-called filter innovation, is the measure used by the Kalman filter to improve the trajectory.

As was mentioned previously, for each pair of registered images, the whole portion of the trajectory that connects them is explicitly corrected, contrary to traditional DSF methods that only explicitly correct the endpoints. For example, all of the robot displacements depicted as dashed blue arrows in Figure 6 will be corrected by the single measurement $z_{k}^{C_{i}}$.

At this point, the standard EKF update equations can be used, which basically depend on the observation function and the measurement vector.

In order to reduce the linearization errors, an iterated EKF (IEKF) [28,29] can be used instead of a classic EKF. Roughly speaking, the IEKF consists of iterating an EKF and relinearizing the system at each iteration, until convergence is achieved. When the IEKF converges, the state vector in the last iteration constitutes the updated state $X_{k}^{+}$.

Section 4 shows and analyzes the results obtained by an implementation of this SLAM approach using an EKF and an IEKF.

## 4. Experimental Results

In order to show the validity of our proposal, some image sequences were recorded in diverse conditions using a simulated and a real robot. Later, our algorithms were run off-line on these recordings.

### 4.1. Experiments in a Simulated Environment

The underwater robot simulator, UWSim [30], was used for the simulated experiments. The environment where the simulated robot was deployed consisted of a mosaic of a real subsea environment. Pictures shown in Figure 3 are examples of the imagery gathered by the simulated underwater camera.

The simulated mission consisted of performing a sweeping task. During the mission execution, images obtained from a monocular bottom-looking camera were gathered. The robot pose was also recorded, but solely used as the ground truth. Altitude was constant in these simulations. The visual odometry was computed in 2D through the homography that transforms image features inter-frames.

The tests were performed with two different keyframe separations, 5 and 10, and using an IEKF instead of an EKF, to minimize linearization errors. With the particular configuration used in these tests, running the algorithm with a separation of five frames means an overlap of 55% of the image between consecutive keyframes in the straight parts of the trajectory. A separation of 10 frames leads to an overlap close to a 10%.

In order to test the robustness of our approach in front of the drift accumulated in the visual odometry estimations, we added synthetic noise to the odometry data. Five noise levels were tested for each keyframe separation. The noise used was additive zero mean Gaussian, and the covariance ranges from a [Σ*_x_*, Σ*_y_*, Σ*_θ_*] = [0,0,0] (Noise Level 1) to [Σ*_x_*, Σ*_y_*, Σ*_θ_*] = [4 × 10^−5^,4 × 10^−5^,5 × 10^−4^] (Noise Level 5). The random noise was added to each visual odometry estimate. For each configuration (5 or 10 frames of separation between keyframes) and noise level, 100 trials were performed in order to obtain significant statistical results. The resulting SLAM trajectories were finally compared to the ground truth in order to quantitatively measure their error. The error of a SLAM trajectory is computed as the mean distance between each of the SLAM estimates and the corresponding ground truth pose.

**Figure 7.**
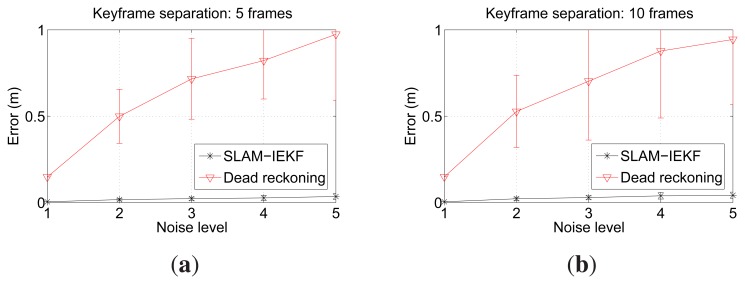
Errors in meters and 2*σ* bound. Noise levels represent the covariance of the synthetic zero-mean Gaussian noise added to odometry, ranging from Noise Level 1 ([Σ*_x_*, Σ*_y_*, Σ*_θ_*] = [0,0,0]) to Noise Level 5 ([Σ*_x_*, Σ*_y_*, Σ*_θ_*] = [4 × 10^−5^, 4 × 10^−5^, 5 × 10^−4^]). (**a**) Using a keyframe separation of five; (**b**) using a keyframe separation of 10.

The results obtained when using a keyframe separation of five are shown in Figure 7a, and those obtained using a keyframe separation of 10 are depicted in Figure 7b. It can be observed that the SLAM error is significantly below the error in dead reckoning. It is clear that the differences due to the keyframe separation and the noise level are very small. Thus, these experiments suggest that our proposal leads to pose estimates whose quality is nearly unrelated to the dead reckoning noise and to the keyframe separation, as long as the overlap between consecutive keyframes is sufficient.

As for the small differences in error when comparing *n* = 5 and *n* = 10, the reason for the overlapping being so different is as follows. The most important pose corrections appear when closing the large loops due to the sweeping trajectory. Of course, if *n* = 5 is used, more candidates are available to perform the loop closure. However, most of these candidates do not provide extra information. That is, due to the proposed registration method, a few candidates are enough to properly close loops. This is consistent with [25], where the authors reduce the computation time by avoiding non-informative poses. In our case, by using *n* = 10, we avoid a significant number of non-informative image registrations.

Furthermore, it is remarkable that the error covariances, which are shown as 2*σ* bounds in Figure 7, are small and significantly lower than those of dead reckoning. That is, even if very different dead reckoning trajectories are used, the SLAM results are very close to the ground truth.

**Figure 8.**
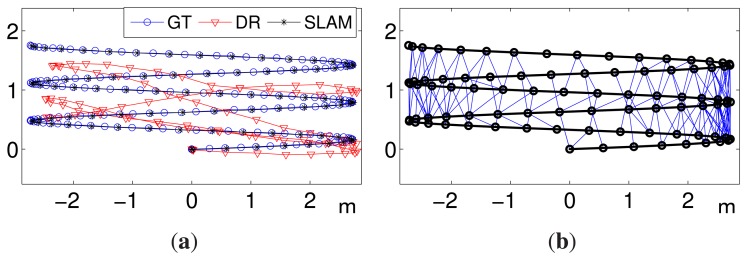
Example of the results obtained with Noise Level 2 and keyframe separation 10. GT and DR denote ground truth and dead reckoning. (**a**) Trajectories; (**b**) registered images.

Figure 8a shows an example of the results obtained with Noise Level 2 and a keyframe separation of 10. The figure shows the resulting SLAM trajectory, which is almost identical to the ground truth. This is especially remarkable since the starting dead reckoning data, as can be seen, are strongly disturbed by noise. Figure 8b depicts the data associations that have been performed during the SLAM operation.

### 4.2. Experiments in a Water Tank

#### 4.2.1. Experimental Setup

**Figure 9.**
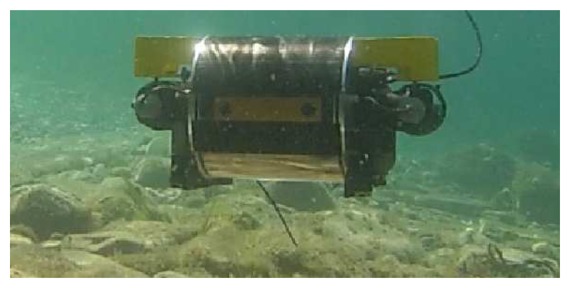
The Fugu-C.

Experiments in aquatic environments were conducted with the Fugu-C platform (Figure 9). Fugu-C is a low-cost, mini-AUV, developed at the University of the Balearic Islands, which can be configured with different sensors. For the specific experiments presented in this paper and to minimize the computation requirements, the following reduced sensor set was used: a down-looking camera gathering monocular video to obtain the (*x*, *y*) odometry, a custom-made altimeter to obtain the distance to the sea bottom (*z*) and a pressure sensor to correct the *z* drift. The computer on board was able to provide both odometric data and height at 10 Hz. The visual odometry was computed by registering pairs of consecutive images using the algorithm described in Section 2. It is important to emphasize that, although this image registration is said to be computationally expensive when used in the SLAM update step, it is not used to perform visual odometry, as it only has to register two images, contrary to the large number of potential candidates when used for SLAM data association.

All of the routes were programmed at a constant depth. The first experiments with the robot were conducted in a pool 7 m long, 4 m wide and 1.5 m depth, whose bottom was covered with a printed digital image of a real seabed. In order to obtain a ground truth in this environment, each gathered image was registered to the whole printed digital image, which was previously known.

**Figure 10.**
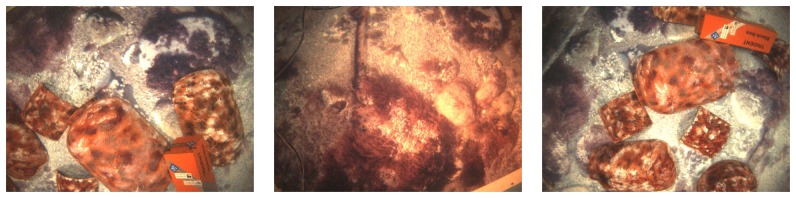
Examples of images obtained during the experiments.

**Figure 11.**
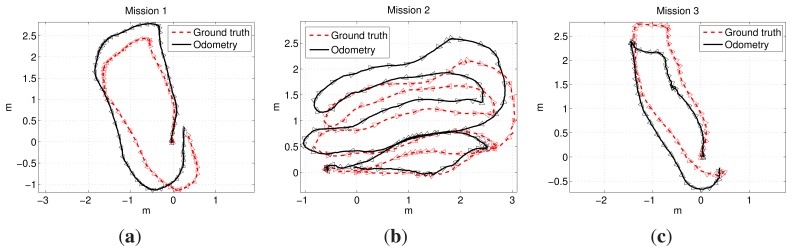
Ground truth and odometry corresponding to: (**a**) the first mission; (**b**) the second mission; and (**c**) the third mission.

In this environment, three missions were executed. The first mission consisted of a single loop. The second mission was a sweeping trajectory, and the third one was also a single loop. However, prior to the execution of the third mission, several objects, such as amphoras and rock replicas, were deployed inside the pool in order to simulate a realistic, non-flat sea floor. Figure 10 shows some examples of the imagery gathered during the third mission. Figure 11a–c shows the ground truth and the visual odometry corresponding to the first, second and third missions, respectively. It can be observed that, although visual odometry properly approximates the overall trajectory, there is also a significant drift error. In these figures, the drift leads to an overall rotated or scaled trajectory with respect to the ground truth, and that is why the odometry end-points are also close to the ground truth end-points. This effect is due to the quality of the visual odometer and the mission length. However, for larger missions or larger odometric errors, this is not likely to happen, as will be shown in further experiments.

#### 4.2.2. Tuning the Search Radius

As stated in Section 3.2.1, deciding which of the gathered images may overlap with the current one is a crucial issue to save execution time. Although RANSAC would reject two non-overlapping images, such a rejection process is time consuming. Thus, it is important to feed RANSAC only images that are likely to overlap and to avoid unnecessary computation.

According to Equation (9), the selection of candidate overlapping images can be performed using a search radius depending on the altitudes at which the images were gathered and a constant parameter R. A simplified version of this criteria assuming constant altitude was also provided in Equation (10).

Tuning *R* ∈ (0, 1] is important to reduce the overall computational cost of the process. Using *R* = 1 should provide the maximum possible quality in the pose estimates, as long as the image registration process successfully discards all wrong image pairings, which is an affordable assumption when using RANSAC to register images. Lower values of *R* should lead to the same quality in the pose estimates with lower computational effort. However, if a too low of a value is chosen for *R*, then some overlapping image pairs will be discarded, and thus, the quality of the pose estimates would decrease. In any case, the computational cost is far below the one obtained without preselecting candidate image pairs.

Accordingly, the optimum value for *R*, *R_opt_*, is the lowest possible value that does not discard image pairs that could be successfully registered by the proposed RANSAC approach. Thus, using *R* = *R_opt_* provides the same quality in the pose estimates as using *R* = 1.

We experimentally selected the *R_opt_* as the one that produces the same pose estimate as using *R* = 1, but with the lowest possible number of false positives. That is, several values of *R* ∈ (0, 1] have been tested, and for each value, the output of each individual pose estimate has been compared with the output obtained when using *R* = 1. Among those values that lead to the same estimate as *R* = 1, the one with the lowest number of false positives has been selected. Deciding if a certain candidate is a false positive or not is straightforward during this tuning process: a false positive is a candidate that is discarded when executing RANSAC.

As a result of this experimental tuning, we found that the optimum values in our experimental setup are *R_opt_* = 0.24 for Mission 1, *R_opt_* = 0.16 for Mission 2 and *R_opt_* = 0.3 for Mission 3. Although these values require *a priori* tuning, the authors would like to emphasize that our proposal is robust in front of values for *R* different from *R_opt_*. For example, using *R* = 0.3 in all three missions would lead to the same pose estimates in all of them and only would slightly increase the computation time in the first two. Using *R* = 0.16 would lead to smaller execution times in Missions 2 and 3, with the negative effect of false negatives in these missions, slightly reducing the quality of the pose estimates. Thus, the experimental tuning provides a value that can be used in similar environments, and in case the maximum quality in the pose estimates is required, *R* = 1 is the best choice.

Table 1 compares the execution time of the three missions using *R_opt_* and *R* = 1. The time was computed executing a MATLAB implementation on an Intel Centrino 2 at 2.4 GHz, with only one CPU kernel used, and running Ubuntu 10.04. The separation between keyframes was 30 frames. It can be observed how using *R_opt_* strongly reduces the execution time in all cases.

It should be noticed that, although the process has been tested using a non-optimized MATLAB implementation executed on a regular computer, the execution time obtained for each mission, when *R_opt_* is used, is close (slightly above) to the real mission duration. For instance, the navigation time for Mission 1 was 169 s, while the whole SLAM process took 186.58 s. Thus, obtaining an on-line version is straightforward.

Next, the quality of the pose estimates is evaluated. We would like to emphasize that these results do not depend on the value of *R_opt_*, as, by definition, using *R* = *R_opt_* leads to the same results as using *R* = 1.

#### 4.2.3. Quantitative Evaluation

The missions already described in Section 4.2.1 were also used to carry out the quantitative analysis: a loop, a sweeping trajectory and a loop over a non-flat terrain. Both ground truth and visual odometry have been shown in Figure 11. In the three cases, a significant odometric error appears.

In order to provide a complete evaluation of the approach, the goal was to compare the quality of every main component of which it is composed.

In our implementation, all of the following combinations were easily configurable and interchangeable, allowing the achievement of the different results exposed later.

First, for each mission, our approach has been tested using IEKF and EKF in the update step. IEKF iterated until convergence was achieved, but a maximum number of 10 iterations was imposed.

For each filter update method, the system has been tested using both the images as they are provided by the camera and filtering them using a Butterworth high pass filter, as suggested in Section 2.

For each of these configurations, three different keyframe separations have been tested: 20 and 30 frames to show the SLAM behavior in a realistic operation and 90 frames to push the system to its limits.

In addition, for each filter update, image treatment and keyframe separation, the visual odometry was corrupted with five different levels of additive zero mean Gaussian error. The covariance of this noise ranged from [Σ*_x_*, Σ*_y_*, Σ*_θ_*] = [0,0,0] (Noise Level 1) to [Σ*_x_*, Σ*_y_*, Σ*_θ_*] = [4 × 10^−5^,4 × 10^−5^,5 × 10^−4^] in Noise Level 5. For each of these cases, 50 trials were executed. This leads to a total of 9000 trials.

The error of each SLAM estimate in every trial was computed by comparing it to the corresponding ground truth pose. The error of each trial is defined as the mean error of the corresponding SLAM estimates. This error was finally divided by the true trajectory length of the corresponding mission, provided by the ground truth. In this way, we measure the meters of error per traveled meter. Thanks to this, the errors obtained for each of the three missions can be compared and also joined in order to obtain an overall measure of quality. Furthermore, if multiplied by 100, this error can be seen as a percentage of error with respect to the trajectory length, and that is how the error is shown in the following figures.

The first relevant result is that, in all of the cases, the statistical differences between keyframe separations of 20 and 30 are barely appreciable. This leads to a similar conclusion to the one obtained under simulation: as long as sufficient overlap between consecutive images is provided, the quality of our proposal is scarcely influenced by the keyframe separation.

**Figure 12.**
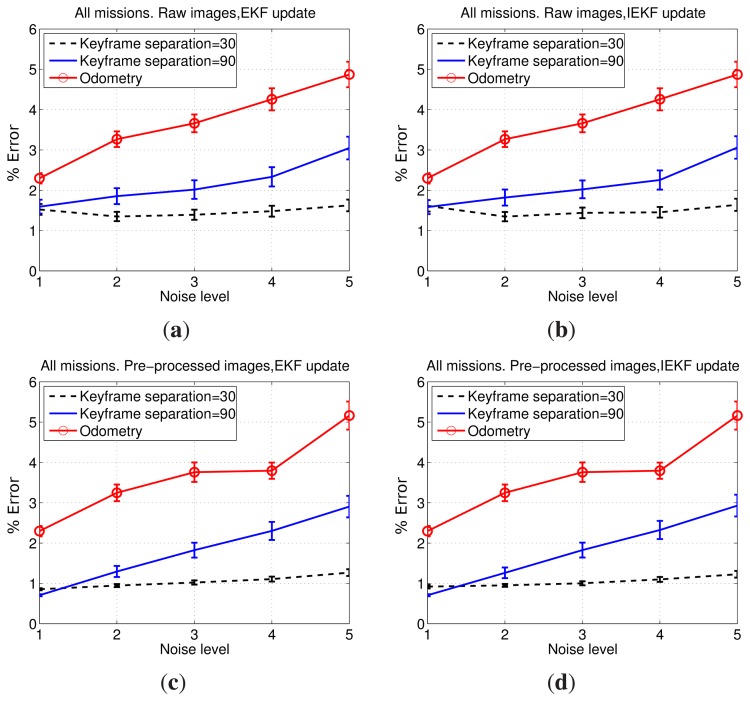
Mean and standard deviation of the errors corresponding to 30 and 90 keyframe separations for: (**a**) raw images and EKF update; (**b**) raw images and IEKF update; (**c**) filtered images and EKF update; and (**d**) filtered images and IEKF update. The standard deviation is depicted as 0.1*σ* to provide a clear representation. Noise levels represent the covariance of the synthetic zero-mean Gaussian noise added to odometry, ranging from Noise Level 1 ([Σ*_x_*, Σ*_y_*, Σ*_θ_*] = [0,0,0]) to Noise Level 5 ([Σ*_x_*, Σ*_y_*, Σ*_θ_*] = [4 × 10^−5^, 4 × 10^−5^, 5 × 10^−4^]).

The results comparing keyframe separations of 30 and 90 are shown in Figure 12. All of the aforementioned test cases are shown. In all four cases, a significant improvement when using 30 frames instead of 90 can be seen. Furthermore, as the noise level increases, the error when using a separation of 30 frames barely increases, whilst using 90 frames leads to a clear error growth. Moreover, the standard deviation of the error remains almost constant when using 30 frames between SLAM executions, suggesting that even large differences between initial estimates, reflected by the large odometric covariance, lead to SLAM results close to the ground truth. Thus, using 30 frames instead of 90 provides a significant improvement in the pose estimates. Accordingly, henceforth, the keyframe separation used during this quantitative evaluation will be 30 frames. However, either using 30 or 90 frames, the SLAM estimates provide an important improvement with respect to the visual odometer.

Figure 12 also provides some insights regarding the other proposed SLAM components. Figure 12a,b shows how the IEKF and the EKF updates provide similar results. The same can be observed comparing Figure 12c,d. This suggests that, at least in these missions, the reduction of linearization errors thanks to the use of IEKF is nearly unobservable. Additionally, when comparing the results corresponding to filtered and non-filtered images, it becomes clear that image filtering actually leads to an appreciable improvement in the accuracy of the pose estimation, particularly when using 30 frames.

**Figure 13.**
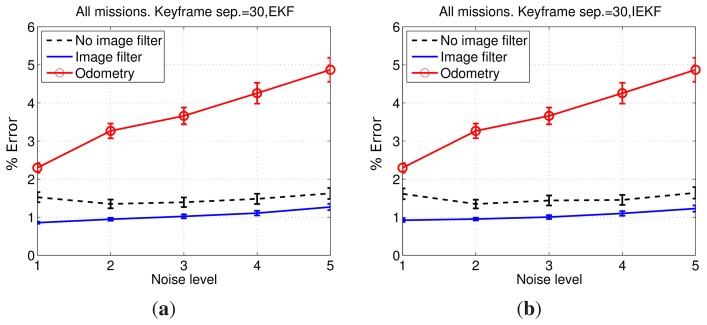
Comparison between pose errors using raw images and filtered images, combined with an (**a**) EKF and (**b**) iterated EKF (IEKF). The standard deviation is depicted as 0.1*σ* to provide a clear representation. Noise levels represent the covariance of the synthetic zero-mean Gaussian noise added to odometry, ranging from Noise Level 1 ([Σ*_x_*, Σ*_y_*, Σ*_θ_*] = [0,0,0]) to Noise Level 5 ([Σ*_x_*, Σ*_y_*, Σ*_θ_*] = [4 × 10^−5^, 4 × 10^−5^, 5 × 10^−4^]).

Figure 13 compares explicitly the errors obtained using raw images and filtered images combined with EKF and IEKF updates. It can be observed that filtering the images actually provides a significant improvement in terms of error reduction with respect to the results obtained using raw images.

As can be observed in the example images shown in Figure 10, they are significantly influenced by the uneven illumination produced by the AUV light source. The proposed image filtering is mainly responsible for alleviating these effects and for enhancing the texture-rich regions. As a result of this, only a few more features appear after filtering, but the resulting descriptors are more discriminative. This increases the number of correct feature matchings and, thus, ameliorates the output of the image registration.

Using this filter in the presence of suspended particles in water may be problematic. In this case, the particles themselves would be enhanced and considered as features. However, as most of the features due to suspended particles would not be consistent with those corresponding to the sea floor, they would be rejected by RANSAC. Anyway, they would, to a greater or lesser extent, have a negative effect on the image registration. In these cases, other image filtering approaches should be considered. However, the uneven illumination conditions are, in our case, more frequent than the existence of suspended particles. Because of this, the described filtering approach is used in this study. The obtained experimental results confirm the benefits of this choice.

Comparing Figures 13a,b confirms that the use of an IEKF barely changes the results. Furthermore, the error standard deviation corresponding to tests conducted with filtered images are smaller than those resulting from the use of non-filtered images.

In summary, the option that combines important reductions in running time with smallest errors in the pose estimates is using a keyframe separation of 30 frames, an EKF for the update step and a previous image filtering to enhance image contrast.

Table 2 summarizes the results by comparing the initial guess provided by the visual odometer and the SLAM output. The percentage of improvement is also shown.

#### 4.2.4. Qualitative Evaluation

**Figure 14.**
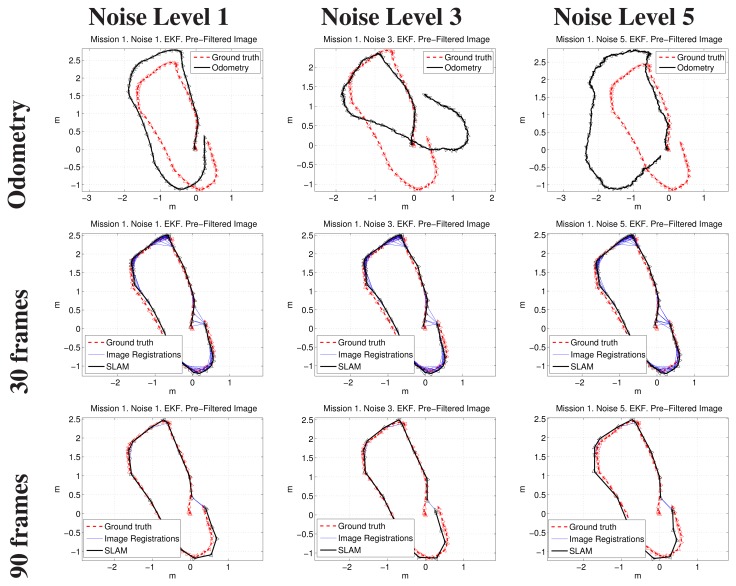
Example results corresponding to the first mission. The first row compares visual odometry and ground truth. The next rows correspond to different keyframe separations. The first, second and third columns are related to Noise Levels 1, 3 and 5, respectively.

**Figure 15.**
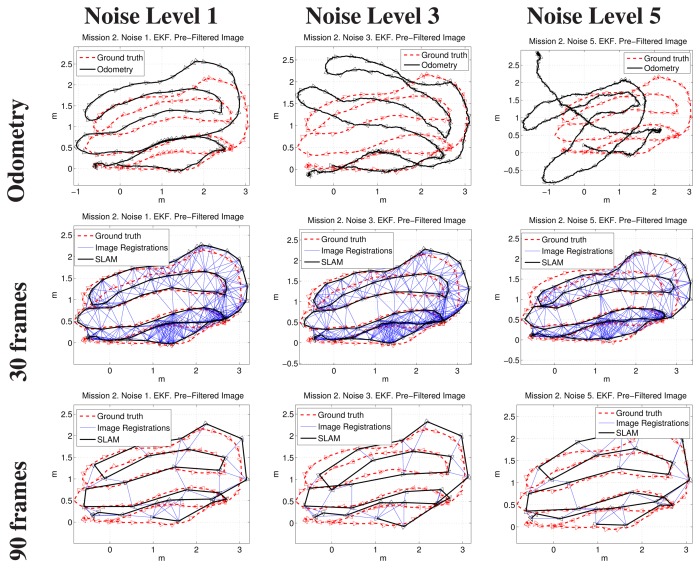
Example results corresponding to the second mission. The first row compares visual odometry and ground truth. The next rows correspond to different keyframe separations. The first, second and third columns are related to Noise Levels 1, 3 and 5, respectively.

**Figure 16.**
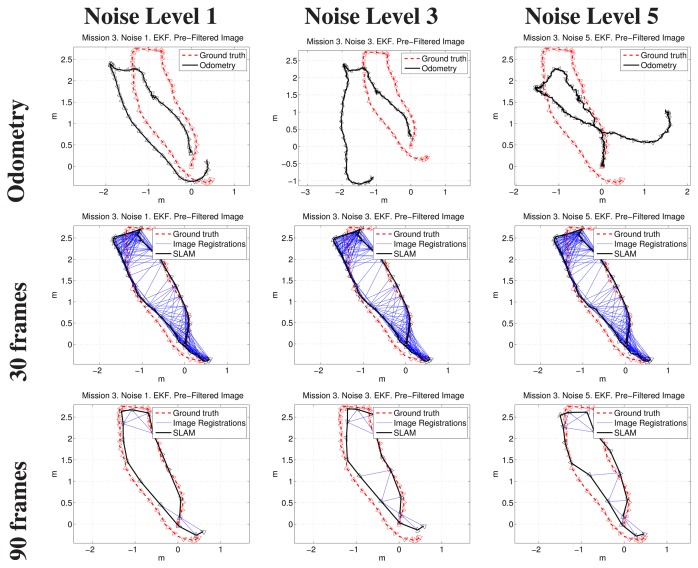
Example results corresponding to the third mission. The first row compares visual odometry and ground truth. The next rows correspond to different keyframe separations. The first, second and third columns are related to Noise Levels 1, 3 and 5, respectively.

Figures 14, 15 and 16 show some representative examples of the SLAM operation under different conditions for the three missions. In all cases, the EKF update and the filtered images were used.

Each figure shows, for its particular mission, the robot trajectory, obtained by composing the odometry and the SLAM pose estimates of executions with 30 and 90 keyframes of separation, with Noise Levels 1, 3 and 5. All of the plots show the positive image registrations in blue and also incorporate the ground truth to facilitate its comparison with the resulting path. The robot is included in the representation as a triangle pointing towards the direction of motion.

It can be observed that, in the three missions, the final results are scarcely influenced by the initial conditions (*i.e.*, the noise level).

### 4.3. Subsea Experiments

**Figure 17.**
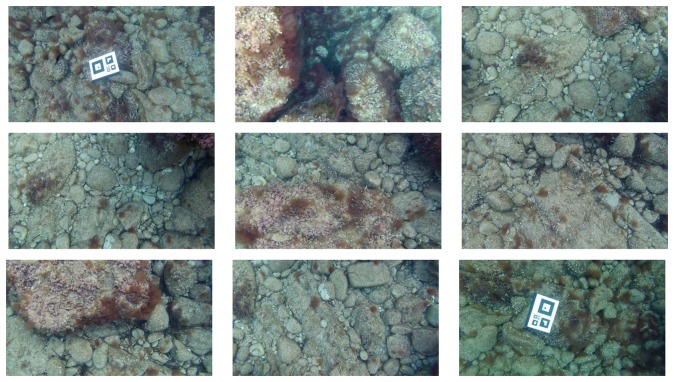
Some images gathered during the experiment in the sea, in Port de Valldemossa. The image on the first row-first column corresponds to the start of the trajectory, and the image on the third row-third column corresponds to the end. The trajectory was performed at a constant depth.

A final experiment was conducted in real undersea conditions, in Port de Valldemossa (Mallorca, Spain). Being a real environment, the floor was non-flat, fully covered by stones and algae, and the robot, which navigated at an approximately constant depth, was influenced by small currents and waves. Furthermore, some minor flickering and shadows appeared in some of the images. Figure 17 shows some examples of the imagery gathered during this experiment. It is worth emphasizing that since the relief of the ground was much lower than the height at which the robot was moving, the system still provided highly plausible results.

Being in a natural environment, a continuous ground truth was unavailable. However, the desired mission was to perform an approximately eight-shaped trajectory with the second loop larger than the first one and ending at the same starting point. An artificial marker was placed on the seabed to assure that the end point of the trajectory corresponded with the initial one.

**Figure 18.**
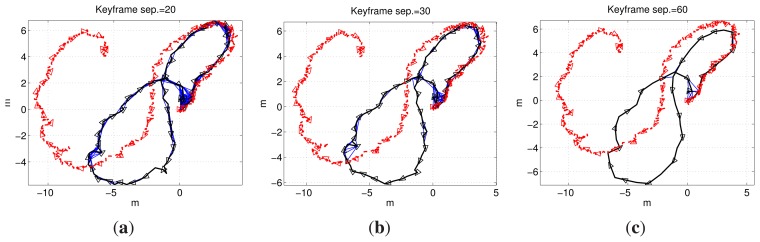
Visual odometry (dashed red line) and SLAM (continuous black like) pose estimates in Port de Valldemossa using keyframe separations of: (**a**) 20 frames; (**b**) 30 frames; and (**c**) 60 frames.

Figure 18 shows the results obtained using a keyframe separation of 20, 30 and 60 frames. All of the plots show the positive image registrations in blue, the trajectory computed from the visual odometry in red and the SLAM trajectory in black. Notice how loop closings are found not only in the origin-end of the trajectory, but also along it. Again, the robot is represented as a triangle, with one of its vertices pointing towards the direction of motion.

It can be observed how the visual odometry presents an important drift in this scenario. To the contrary, the SLAM estimates are much closer to the real trajectory, and thanks to several loop closings established during the mission execution, the trajectory is considerably correct, ending at the same point where it started.

## 5. Conclusions and Future Work

This paper proposes a practical approach to perform underwater visual localization, which improves the traditional EKF-SLAM by reducing both the computational requirements and the linearization errors. Moreover, the focus of this paper is the image registration, which is used in the SLAM data association step, making it possible to close loops robustly. Thanks to that, as shown in the experiments, the presented approach provides accurate pose estimates, both using a simulated robot and a real one, in controlled and in real underwater scenarios.

Nonetheless, the presented approach makes two assumptions that limit the environments where the robot can be deployed. On the one hand, it is assumed that the camera is always pointing downwards nadir the sea floor. Although this may seem a hard requirement, the experiments with the real robot suggest that the small oscillations in roll and pitch inherent to the robot motion are not significantly influencing the results of our approach. However, avoiding this requirement is one of our future research lines. The simplest way to solve this problem is to use the roll and pitch provided by the gyroscopes in the IMU and to use this information to re-project the feature coordinates. On the other hand, the proposal assumes a locally flat floor. Even so, some experiments included in this paper suggest that real oceanic floors with limited relief are well tolerated by our method. However, upcoming work is currently focused on using pure stereo data to overcome this restriction and to perform 3D SLAM with six DOF.

Finally, the execution time depends on the appropriate selection of *R_opt_*. Although the obtained values constitute good initial guesses for all of those missions that have to be executed under similar conditions, improving this aspect is also a line of future research. Our proposal in this aspect is to make the search radius automatically change on-line, depending on the RANSAC failures.

## Figures and Tables

**Table 1.** Execution time comparison using *R* = *R_opt_* and *R* = 1.

**Mission**	***R*=*R****_opt_*	**R=1**	**Improvement**
1	186.58 s	726.84 s	74.33%
2	416.35 s	2243.74 s	81.44%
3	143.54 s	479.57 s	70.07%

**Table 2.** Comparison of errors in visual odometry and SLAM using a keyframe separation of 30, EKF update and filtered images. Errors are expressed as the percentage of error with respect to the trajectory length.

**Noise Level**	**1**	**2**	**3**	**4**	**5**
Visual odometry	2.3%	3.3%	3.7%	4.3%	4.9%
SLAM	0.8%	0.9%	1.0%	1.1%	1.3%
Improvement	62.8%	71.0%	72.1%	74.0%	74.0%
